# MultiRankSeq: Multiperspective Approach for RNAseq Differential Expression Analysis and Quality Control

**DOI:** 10.1155/2014/248090

**Published:** 2014-05-27

**Authors:** Yan Guo, Shilin Zhao, Fei Ye, Quanhu Sheng, Yu Shyr

**Affiliations:** Center for Quantitative Sciences, Vanderbilt University, Nashville, TN 37027, USA

## Abstract

*Background*. After a decade of microarray technology dominating the field of high-throughput gene expression profiling, the introduction of RNAseq has revolutionized gene expression research. While RNAseq provides more abundant information than microarray, its analysis has proved considerably more complicated. To date, no consensus has been reached on the best approach for RNAseq-based differential expression analysis. Not surprisingly, different studies have drawn different conclusions as to the best approach to identify differentially expressed genes based upon their own criteria and scenarios considered. Furthermore, the lack of effective quality control may lead to misleading results interpretation and erroneous conclusions. To solve these aforementioned problems, we propose a simple yet safe and practical rank-sum approach for RNAseq-based differential gene expression analysis named MultiRankSeq. MultiRankSeq first performs quality control assessment. For data meeting the quality control criteria, MultiRankSeq compares the study groups using several of the most commonly applied analytical methods and combines their results to generate a new rank-sum interpretation. MultiRankSeq provides a unique analysis approach to RNAseq differential expression analysis. MultiRankSeq is written in R, and it is easily applicable. Detailed graphical and tabular analysis reports can be generated with a single command line.

## 1. Introduction

Gene expression refers to the appearance of a characteristic or effect in the phenotype that can be attributed to a particular gene. The development of microarray technologies has helped biomedical researchers make significant advances in the last decade by allowing high-throughput gene expression screening on all known genes. The introduction of RNAseq technology further revolutionized the field of gene expression research with accurate measurements of transcripts instead of estimating relative measures and with the detection of structural variants such as splicing and gene fusion. RNAseq uses next-generation sequencing (NGS) technologies to sequence cDNA that has been reverse transcribed from RNA. It is commonly believed to be superior to microarray technology due to its ability to quantify gene expression at higher resolution (exon and CDS level) and detect structural variations. As early as 2008 [1], RNAseq has been hailed as the eventual replacement of microarray technology, and since then, multiple studies [2–6] have also illustrated the advantages of RNAseq and come to similar conclusions by analyzing real data or through thorough simulation study.

RNAseq technology introduces new and exciting opportunities to researchers in the field of biomedical research as well as stiff analysis challenges for bioinformaticians. The rich genomic information RNAseq technology contains gives RNAseq the decisive advantage over microarray but adds complication in the analysis phase. Several unique characteristics contribute to the difficulty of RNAseq data analysis. First, in RNAseq, the expression values are usually directly represented by the number of reads or adjusted number of reads aligned to a gene. For a nonexpressed gene, zero reads are aligned to the gene's genomic span. Because microarray technology is based on fluorescence intensity, there is always a nonzero background intensity, allowing microarray data to be log-transformed. In contrast, due to the large number of zeros for nonexpressed genes in RNAseq data (often around 50%), log transformation results in many invalid mathematical operations. The typical range of an RNAseq dataset is huge, between 0 and 10,000+ compared to microarray's 2 to 15 after RMA normalization (with log 2 transformation). Because RNAseq's expression value starts from 0, large fold change can result from two very small expression values. For example, the fold change between 0.1 and 0.001 is 100, but both 0.1 and 0.001 should be considered nonexpressed. In addition, there are many sequencing and alignment artifact that can skew RNAseq data such as errors from demultiplexing and alignment ambiguity caused by highly homologous genomic regions.

These bioinformatic challenges create difficulty for RNAseq data analysis. In this study, we focus on the inconsistency of differential expression analyses and the lack of multiperspective quality control. First and foremost, to date the research community has yet to come to a consensus on the best of a multitude of different approaches for differential gene expression analysis of RNAseq data. The pioneer of RNAseq differential expression analysis, Cufflinks, is based on reads per kilobase per million mapped reads (RPKM) [7] and fragments per kilobase of transcript per million mapped reads (FPKM) [8]. A similar approach is RNAseq by expectation-maximization (RSEM) [9]. RPKM, FPKM, and RSEM can be classified as read normalization-based methods.

Another type of RNAseq differential gene expression analysis is based on read count. Many read count-based methods have been developed including DESeq [10], DEGseq [11], edgeR [12], baySeq [13], TSPM [14], NBPSeq [15], SAMseq [16], and NOIseq [17]. Since they are dealing with RNAseq count data, the majority of them are based on Poisson or negative binomial distributions. But there are implementation details that separate them. For example, edgeR moderates dispersion estimates toward a trended mean, whereas DESeq takes the maximum of the individual dispersion estimates and the dispersion-mean trend, and baySeq uses an empirical Bayes approach assuming a negative binomial distribution of the data. Several studies [4, 18–21] have attempted to evaluate different normalization and differential gene comparison methods for RNAseq data. Although no final conclusion can be reached, through simulation analysis of real data, it has been found by multiple sources that DESeq, edgeR, and baySeq were able to maintain a reasonable false-positive rate without any loss of power. More recently, nonparametric approaches, such as SAMseq and NOIseq, were proposed aiming to overcome limitations of aforementioned parametric as they can be influenced by “outliers” in the data. In this paper, we focus on the currently widely applied parametric methods for RNAseq gene expression analysis, but it is easy to incorporate other methods including nonparametric approaches in MultiRankSeq.

In addition to the lack of consensus on the best statistical method, another issue associated with RNAseq data analysis is the lack of complete quality control. The majority of high-throughput sequencing quality control tools were designed exclusively for raw data. Previously, we have proposed a three-stage quality control [22] strategy for exome sequencing analysis that emphasizes the need to implement quality control at all stages of exome sequencing processing: raw data, alignment, and variant calling. The same idea can be easily adapted to the three stages of RNAseq analysis as well: raw data, expression quantification, and differential expression analysis. There have been several tools designed for RNAseq quality control such as RNA-SeQC [23] and RSeQC [24]. These tools generally target the raw data and expression quantification steps by calculating quality control parameters such as read coverage, and GC bias. However, quality control on differential expression analysis is often not considered.

In this paper, we propose a multimethod rank-sum approach for RNAseq expression analysis that combines multiple RNAseq differential expression analysis packages. Combining multiple methods of RNAseq data analysis has been previously suggested. For example, Robles et al. suggested that using a combination of multiple packages may overcome the possible bias susceptibility of a given package to a particular dataset of interest [20]. In another study by Soneson et al., the authors suggested the use of transformation-based approaches (the variance stabilizing transformation provided in the DESeq R package and the voom transformation from the limma R package) combined with LIMMA [25], which performed well under many conditions. In this study, we present a tool, MultiRankSeq, for RNAseq differential gene expression analysis. This tool offers rank-sum-based differential gene expression analysis, comprehensive diagnostic quality control assessment, and automated graphical reports. The input of MultiRankSeq is a read-count matrix. MultiRankSeq is implemented in R, and it is freely available for public use. MultiRankSeq can be downloaded from https://github.com/slzhao/MultiRankSeq.

## 2. Materials and Methods

Differential expression analysis can only be conducted between two phenotypes such as tumor versus normal or treated versus untreated. The ideal assumption for conducting differential expression analysis is that gene expression patterns are similar for samples within the same phenotype group (i.e., relatively homogeneous). Sometimes, however, this assumption does not hold true. A sample from one phenotype group may be more similar to the samples from the other phenotype groups based merely on expression profile. Unfortunately, the homogeneity of gene expression patterns within the same group is not always checked before conducting differential gene expression analysis. One simple yet effective way to check this assumption is through cluster analysis. Clustering refers to the task of grouping together a set of samples with similar gene expression patterns. To determine the pairwise sample gene expression pattern similarity, a similarity or distance measurement must be employed. In MultiRankSeq, we chose to use Spearman's correlation coefficient. Because the input of MultiRankSeq is read count, Spearman's correlation coefficient is used as it is more robust to handle skewness and outliers than a parametric method. MultiRankSeq performs unsupervised clustering using all genes to best represent the raw expression pattern of each sample. Samples clustered outside the true phenotype group are considered to be misclassified. This could occur due to sample contamination or other technical reasons. If the majority of the genes lack variation among samples, the cluster may be unrepresentative of the true phenotype group. To alleviate this, MultiRankSeq performs additional cluster analyses on read counts filtered by the top 5% and 10% coefficient of variation. In theory, the clustering should improve as more stringent coefficient of variation cutoffs is used.

MultiRankSeq performs a gene expression integrity check by drawing the read count distribution and the normalized read count distribution. Normalization is done by dividing each gene's read count by the total read count of all genes in this sample. One of the unique optional features offered by MultiRankSeq is the ability to detect batch effect. Batch effects can be a problem with RNAseq data [26]. The most common sequencing failures often occur nonrandomly by lane, flow cell, run, or machine. MultiRankSeq recognizes and records the machine name, run ID, flow cell ID, and lane ID of an experiment from either the FASTQ file or BAM file. Based on this information, MultiRankSeq determines whether batch effect exists using the nonparametric Kruskal-Wallis [27] test and Fligner-Killeen test of homogeneity of variances [28]. MultiRankSeq uses boxplots and correlation matrices to demonstrate the expression variation between samples.

The idea behind MultiRankSeq's algorithm for integrating the results from multiple RNAseq analysis tools is based on the same analytic principle as the weighted flexible compound covariate method (WFCCM) [29]. WFCCM was designed to integrate the findings of multiple analysis methods (e.g., Kruskal-Wallis test, Fisher's exact test, permutation *t*-test, SAM, WGA, and modified info score) to identify the most significant gene expression associated with biological status and thereby allow for class-prediction modeling based on differential gene expression. In other words, WFCCM extends the compound covariate method by allowing for more than one statistical analysis method to be considered in the covariate and reduces the dimensionality of an analytic problem by generating a single covariate calculated as a weighted sum of the class predictors identified as most important.

Based on previous studies [18–21] and our own evaluation [30], we selected three methods for MultiRankSeq—DESeq, edgeR, and baySeq—and combined their algorithms in MultiRankSeq. The tabular report provided by MultiRankSeq includes log 2 fold change, raw *P*-value, and false discovery rate (FDR) adjusted *P* value from all three methods except for baySeq because the Bayesian-based method does not calculate fold change. We rank the genes based on the raw *P* value rather than FDR-adjusted *P* value because the latter often has a large number of tied values. The sum of the rankings from all three methods is reported in the last column of the tabular report to serve as an overall ranking of genes. The sum of ranks can be used as a confidence level of differential expression. The smallest rank sums indicate differentially expressed genes are consistent among the three methods.

MultiRankSeq provides concordance analysis of the results from the three methods and detailed visualizations using various figures such as Venn diagram, heatmap, and scalable volcano plot to summarize and illustrate the analysis results. Venn diagrams demonstrate the logical relations between the three methods based on parameters such as fold change, adjusted *P* value, and top ranked genes. The heatmap is used for visualization of gene expression patterns in a color scale. The correlation scatter plots depict the general consistency between the methods. The scalable volcano plot can help the user visualize the genes based on fold change, *P* value, and ranking simultaneously. The rank of the gene is reflected by the size of the corresponding dot on the volcano plot.

## 3. Result and Discussion

### 3.1. Results

We demonstrate MultiRankSeq using two example datasets from the TCGA breast cancer and performed analysis using MultiRankSeq V1.1.2. This version of MultiRankSeq uses edgeR 3.4.2, DESeq 1.14.0, and baySeq 1.16.0 as the primary three differential expression analysis packages. The first example dataset contains RNAseq data from 3 tumors and 3 adjacent normal tissues from same patients (TCGA-A7-A0D9, TCGA-BH-A0B3, and TCGA-BH-A0BJ). This example is used to show the MultiRankSeq's cluster functionality.

**Figure 1:**
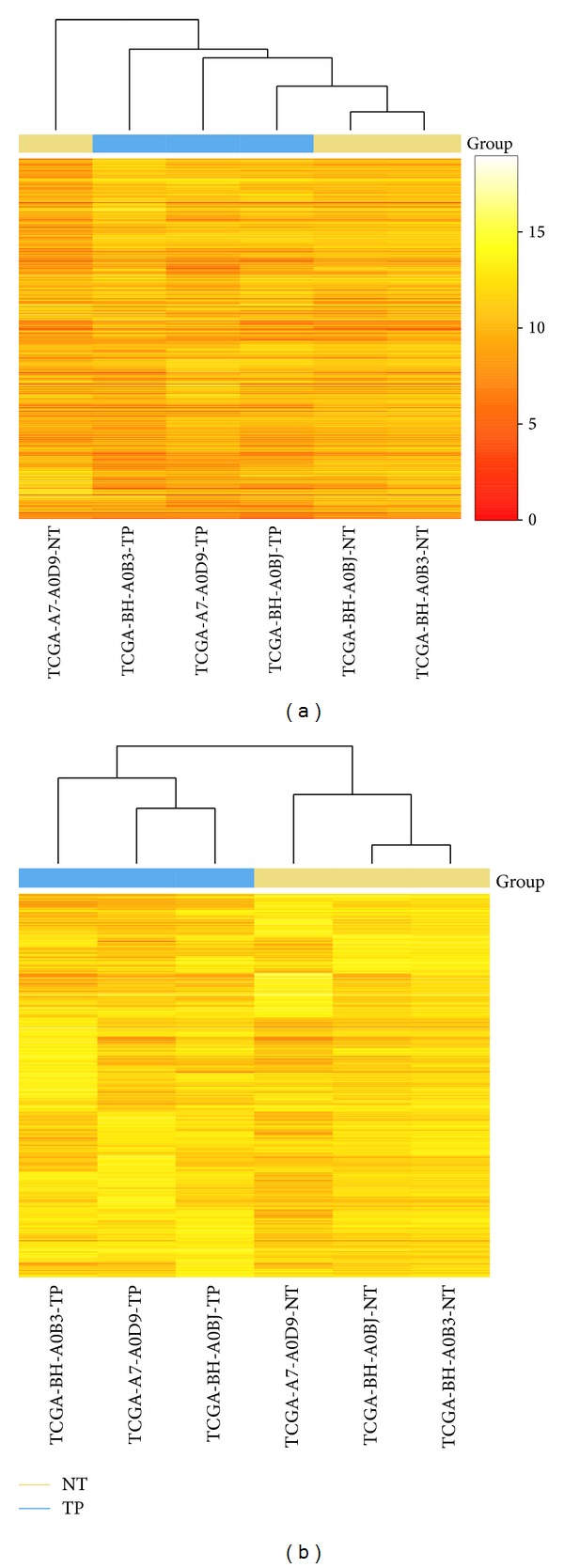
(a) Cluster result using all genes shows control 1 clustered together with disease group. (b) Cluster results using genes with top 5% coefficient of variation, control group, and disease group are now clustered correctly.

**Figure 2:**
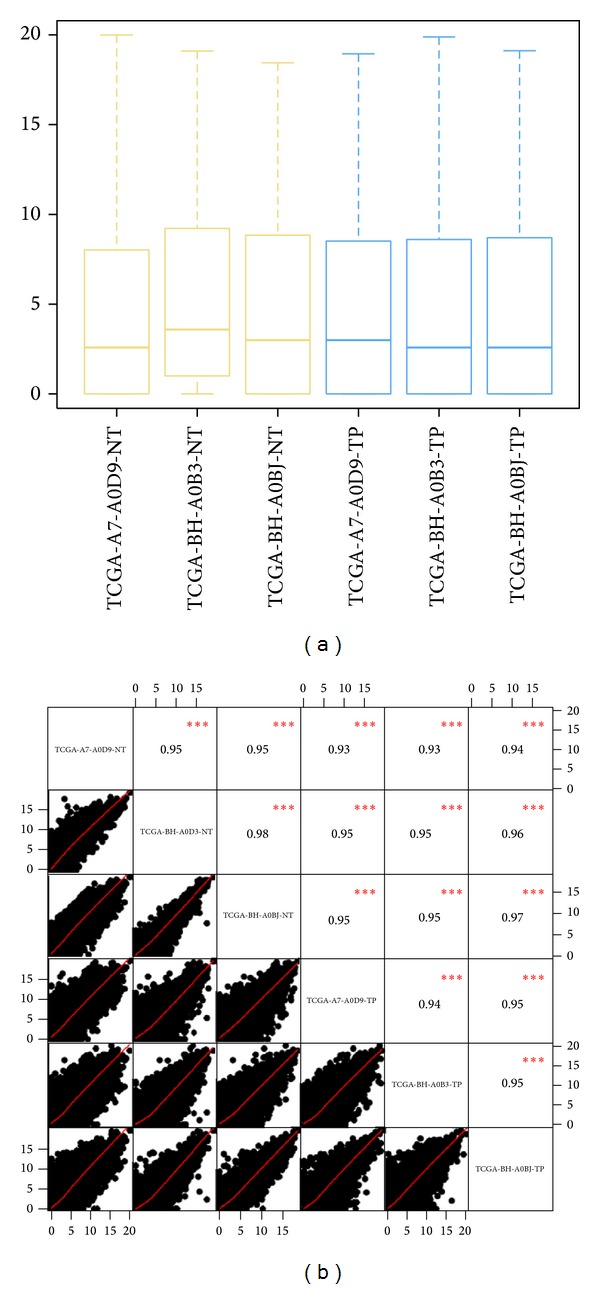
(a) Boxplots of gene raw read count. (b) Correlation matrix of all genes between all pairs of samples using raw read count.

When using unfiltered data, an adjacent normal sample was clustered with the tumor group (Figure 1(a)). Normally, we may consider this sample problematic and remove it from the analysis; however, cluster result using genes with the top 5% coefficient of variation showed the correct grouping (Figure 1(b)). The misclassified sample in Figure 1(a) is likely due to noise caused by genes that lack variation among samples. Therefore, part of its information can be used for the analysis instead of completely removing the sample.  Figure 2 shows additional quality control matrix produced based on the example of dataset 1.

The second example dataset also contains RNAseq data from 3 tumors and 3 adjacent normal tissues from same patients (TCGA-BH-A0BM, TCGA-BH-A0C0, and TCGA-BH-A0DK). Using this example, we demonstrate the complete MultiRankSeq's functionality.

**Figure 3:**
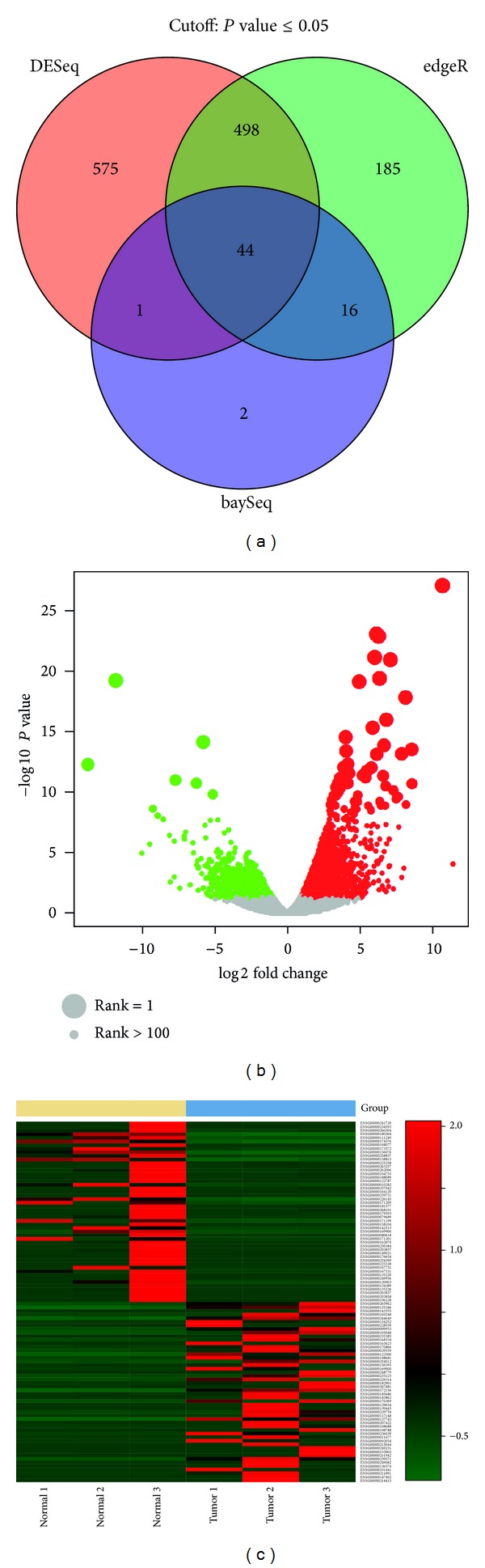
(a) Venn diagram of differential expression analyses by DESeq, edgeR, and baySeq. The Venn diagram can be drawn based on *P* value, fold change, or rank. (b) Scalable volcano plot representing fold change, *P* value, and rank. Rank is presented as the size of the circle, and larger size denotes higher ranking. (c) Heatmap of top differentially expressed genes. MultiRankSeq produces heatmap based on *P* value, fold change, and rank; only genes selected by fold change are shown here.

The example result figure produced by MultiRankseq using this example can be seen in Figure 3. The full HTML reports of MultiRankSeq from the example data can be found at the tool's hosting website. The complete R command used to generate the results can be viewed as follows:

 library(MultiRankSeq);
 #Load the downloaded data into R, and generate group definition;
 
Figure 1<read.csv(“TcgaFigure 1.csv”,header=T,row.names=1,check.names=F);
 
Figure 3<read.csv(“TcgaFigure 3.csv”,header=T,row.names=1,check.names=F);
 group=c(0,0,0,1,1,1);
 #Generate report;
 reportF1<-MultiRankSeqReport; (output=“reportFigure 1.html”,rawCounts=TcgaFigure 1, group=group);
 reportF3<-MultiRankSeqReport; (output=“reportFigure 3.html”,rawCounts=TcgaFigure 3, group=group).

Even though, majority of the time, DESeq, edgeR, and baySeq agree with each other, there is still occasional disagreement. This can be demonstrated through the second example. We observed that when there is a large variation in the read counts, especially when the homogeneity assumption seems to be violated, the 3 methods can disagree with each other significantly. One particular example is the gene IGHG2 (Table 1). Based on the FDR-adjusted *P* value, only edgeR considered it to be significant. The FDR-adjusted *P* values were 0.047, 0.28, and 0.91, respectively, for edgeR, DESeq, and baySeq. We then performed an additional analysis using Cuffdiff [31]. Cuffdiff agreed with edgeR with an FDR-adjusted *P* value <0.001. The log 2 fold changes of IGHG2 produced also spans a large range (from 2.92 to 5.83). After adjusting for the total number of reads, the variation becomes less obvious with each of the methods (Table 2). However, in this particular case, edgeR seems to have performed more effective variation stabilization.

In terms of number of winner genes (adjusted *P* < 0.05) identified, the three methods differ hugely in example 2 (DESeq = 1118, edgeR = 743, and baySeq = 63). We performed network, pathway, and biological functionality analysis using ingenuity. The results are split into seven categories: genes identified by DESeq, edgeR, baySeq, singleton genes that identified each of the three methods, and overlapped genes among the three methods. Singleton gene means this gene is only identified by one method. The top five networks, biological functionalities, and canonical pathways are reported (see Table S1 in Supplementary Material available online at http://dx.doi.org/10.1155/2014/248090). The functionality results of DESeq and edgeR are similar because they have large overlap; the results of baySeq are more unique because of less overlap with other methods.

### 3.2. Discussion

Performing traditional comparative statistical analysis methods such as *t*-test or Wilcoxon rank-sum test requires at least 3 samples for variation to generate meaningful results. Such limitations also apply to DESeq, edgeR, and baySeq. Cuffdiff, however, can assign *P* values even for 1 sample versus 1 sample. In order to do this, it makes the assumption that similarly expressed genes have similar variance and the majority of the genes are not differentially expressed. In many studies, these assumptions will hold true, but including multiple samples in a group will always generate more robust results. The current version of MultiRankSeq only considers methods based on read count data. However, it is our goal to incorporate Cuffdiff in the future research.

RNAseq data is difficult to analyze and sometimes is methodology-dependent as previously discussed. MultiRankSeq tackles this problem from a different perspective by combining ranked results from multiple well-rated RNAseq analysis methods. This approach brings more confidence to the selection of truly differentially expressed genes. Another novelty that MultiRankSeq brings is the bridging of the gap between quality control and statistical analysis. The report generated by MultiRankSeq is comprehensive and helps the user better appreciate the power and complexity of RNAseq data. MultiRankSeq is based on an intuitive idea of combining multiple methods yet very practical. Because MultiRankSeq is designed with user friendliness and flexibility in mind, additional RNAseq analysis programs can be easily added to it in the future if needed. In conclusion, MultiRankSeq is a simple framework of RNAseq data analysis which provides tremendous convenience and an alternative perspective for researchers who conduct routine RNAseq analysis.

We do not claim that combining the results of multiple methods will always produce more accurate result. There are the scenarios when the minority method is corrected. Thus combining three methods may still produce false positive results. However, if multiple methods agree, the probability of generating the true positive results will most likely to increase. The goal of MultiRankSeq is to provide the user with higher confidence to pick a gene that is significantly differentially expressed with high probability.

## Supplementary Material

Supplementary Table S1: Summary of Intenuity pathway analysis results.

## Figures and Tables

**Table 1:** Analysis difference for IGHG2.

Method	Adjusted *P* value	log⁡2 FC	Rank
DESeq	0.278	3.00	2572
edgeR	0.047	2.92	712
baySeq	0.907	NA	24962
Cuffdiff	<0.001	5.83	13

**Table 2:** Read count of samples for IGHG2 gene.

	Disease 1	Disease 2	Disease 3	Control 1	Control 2	Control 3
Read count (IGHG2)	391	2038	338	634	10282	1764
Total read count	49870084	65550902	71454121	35641084	44863975	49052840
Adjusted read Count^1^	78	311	47	178	2292	360

^1^Adjusted read count of gene A is computed as read count of a gene A divided by total read count of the sample times a constant.
